# A rare presentation of soft tissue chondroma: A case report

**DOI:** 10.1016/j.ijscr.2020.06.045

**Published:** 2020-06-13

**Authors:** Fahmi H. Kakamad, Abdulwahid M. Salih, Marwan N. hassan, Shvan H. Mohammed, Ari M. Abdullah

**Affiliations:** a College of Medicine, Department of Cardiothoracic and Vascular Surgery, University of Sulaimani, Sulaimani, Kurdistan, Iraq; b Smart Health Tower, François Mitterrand Street, Sulaimani, Kurdistan, Iraq; c Kscien Organization, Hamdi Str, Azadi Mall, Sulaimani, Kurdistan, Iraq; d College of Medicine, Department of Surgery, University of Sulaimani, Sulaimani, Kurdistan, Iraq; e Undergraduate Student. College of Medicine, University of Sulaimani, Sulaimani, Kurdistan, Iraq; f Sulaimani Teaching Hospital. Sulaimani, Kurdistan, Iraq

**Keywords:** Soft tissue chondroma, Extraskeletal chondroma, Benign

## Abstract

**Introduction:**

Extraskeletal chondroma (ESC) is a benign, slow-growing cartilaginous tumor arising from tenosynovial sheaths. The aim of this article is to report and discuss a case of ESC affecting upper thigh.

**Case report:**

A 41-year-old male presented with a swelling in the medial aspect of the left thigh. On clinical examination, there was a 10 × 15 cm non-tender, hard, ill-defined mass in the medial aspect of the left upper thigh. Ultrasound showed a large well defined thick wall mass, located inside gracillis or adductor muscles. Magnetic resonance imaging showed a large well defined mass involving the adductor compartment of the upper thigh. The patient underwent wide local excision under spinal anesthesia. The histopathological examination of the specimen revealed binucleated chondrocytes with dots of calcification confirming ESC.

**Discussion:**

There are many theories trying to explain the origin of ESC, as some authors think that it originates from the pluripotent cells of the tenosynovium, while others state that it may be derived from metaplasia of the tendon sheath. In this case, the lesion was completely surrounded by muscle fibers away from the nearby tendons.

**Conclusion:**

Extraskeletal chondroma is a rare benign lesion, although mostly affect the upper extremities, it can be found anywhere in the body, histopathological examination of the specimen is the diagnostic method of choice.

## Highlights

• Extraskeletal chondroma is a benign, slow-growing cartilaginous tumor arising from tenosynovial sheaths.
• It may present challenges for the treating physician.
• This article reports and discusses a case of extraskeletal chondroma affecting upper thigh.

## 1 Introduction

Chondromas are described as benign cartilaginous tumors. They can be found in any part of the body with cartilaginous bones, but often occur in short tubular bones, especially metacarpals and phalanges [1]. A benign, slow-growing cartilaginous tumor arising from tenosynovial sheaths is called an extraskeletal chondroma, or a soft tissue chondroma, in another word, it is the soft tissue chondroma adjacent to tendons without connection to bone or periosteum [2]. ESC arises from all tissues except bone or cartilage [3]. The peak age of affection is between the third and sixth decades of life without sex preference [4]. To date, only a few cases of ESC have been reported in the lower limbs [5].

The aim of this article is to report and discuss a case of ESC affecting upper thigh in line with SCARE guidelines with brief literature review [6].

### 1.1 Patient information

A 41-year-old male presented with a swelling in the medial aspect of the left thigh. His past medical and surgical history was clear. He was neither smoker nor alcoholic. There was no family history of the current situation.

### 1.2 Clinical examination

There was a 10 × 15 cm non-tender, hard, ill-defined mass in the medial aspect of the left upper thigh extending to the inguinal region. No skin changes, no neurovascular abnormality.

### 1.3 Diagnostic assessment

**Fig. 1:**
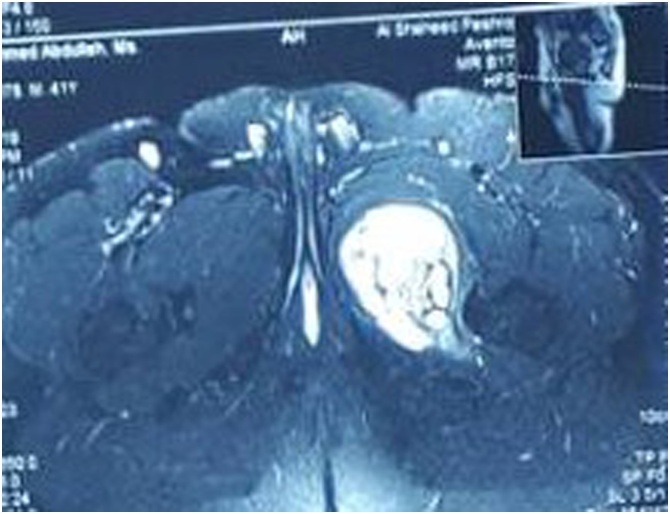
Magnetic resonance imaging (T2 weighted, axial section) showing hyperintense mass lesion involving the adductor compartment of the left upper thigh.

**Fig. 2:**
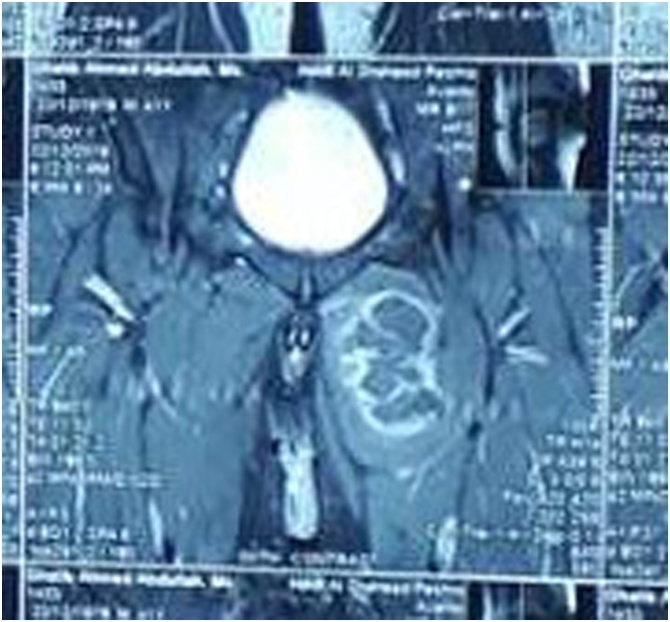
Magnetic resonance imaging (T1 weighted, coronal section) showing isointense mass lesion with peripheral enhancement involving the adductor compartment of the left upper thigh.

Hematological tests were normal. Ultrasound showed that the mascular layers of the upper medial compartment of the left thigh contained a large well defined thick wall mass, 10 × 7.5 cm in size, located inside gracillis or adductor muscles, the features were suspicious for hemangioma. Magnetic resonance imaging (MRI) showed a large well defined mass involving the adductor compartment of the upper thigh extending to the lower part of the gluteal region. The lesion was hyperintense to the surrounding muscles on T2-weighted image (Fig. 1) and isointense to them with peripheral enhancement on T1-weighted image (Fig. 2). Core needle biopsy was inconclusive.

### 1.4 Therapeutic intervention

**Fig. 3:**
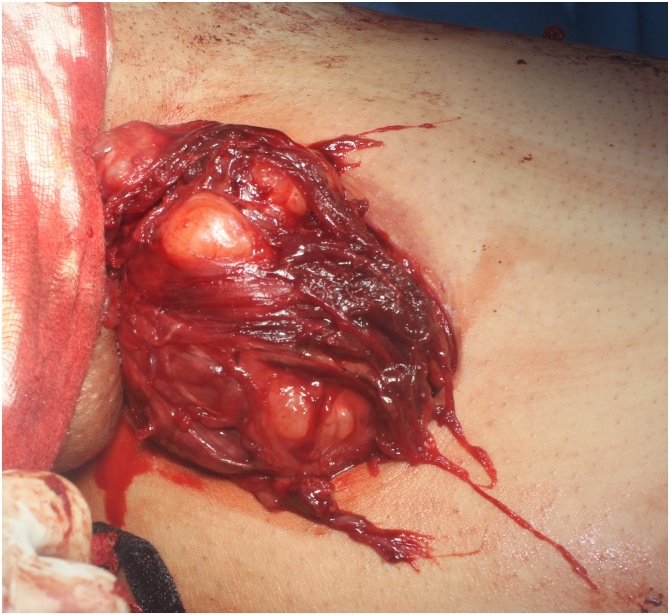
Intraoperative findings of the lesion.

**Fig. 4:**
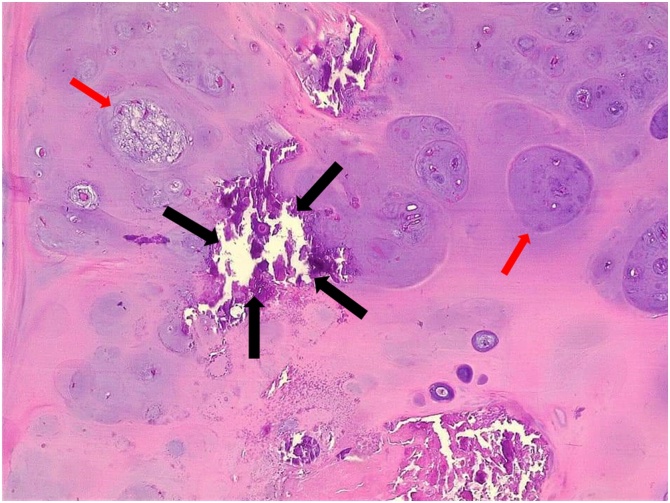
Photomicrography of chondroma with Hematoxylin and Eosin (H&E) stain showing mature hyaline cartilage (red arrows) with multiple foci of calcification (black arrows).

Under the supervision of the first author with spinal anesthesia, in supine position, the patient underwent wide local excision through a longitudinal incision in the medial aspect of the left upper thigh (Fig. 3). The histopathological examination of the specimen revealed a well demarcated mass with lobulated border pushing the margins, composing of mature hyaline cartilage without marked atypia, mitoses or necrosis, associated with multiple foci of calcification, the features were consistent with ESC (Fig. 4).

### 1.5 Follow up

The postoperative course was uneventful. The patient remained overnight in hospital. He was discharged on simple oral analgesic. Three months later, he was found to be healthy.

## 2 Discussion

Although chondroma is a common tumor, ESC is an exceptionally rare benign lesion that arises from soft tissue, without continuity to the bone or periosteum [7]. The common site for ESCs is the upper limbs (72%) especially the hands [8]. In the current case, ESC affected the medial aspect of the left thigh, to best of our knowledge, this is the first time to be reported in this region.

There are many theories trying to explain the origin of ESC, as some authors think that it originates from the pluripotent cell of the tenosynovium, while others state that it may be derived from metaplasia of the tendon sheath [4]. In this case, the lesion was completely surrounded by muscle fibers away from the nearby tendons.

In the literature, the lesion has been described as a well demarcated one, sometimes lobulated and one to two centimeters in size [9]. It is a slowly growing, painless, single or multiple mass [7]. Peters et al. reported a case of ESC with an usual presentation of rapid growth with intractable pain [10]. Although this lesion had a painless, slowly growing features and well-defined border with a lobulated surface, the size was much larger (about 10–15 cm) than been described previously.

Ultrasound examination is usually the starting point in work up of swelling, however MRI is the method of choice for evaluation of ESC. It defines the contour, the extent, the shape, calcification and the relation of the tumor to the surrounding structures. Sometime FNAC or core needle biopsy is required to determine the exact diagnosis preoperatively, especially when the physician worried about malignancy [11]. Regarding the current case, being large size with completely embedded in the muscle fibers made the sonographer think of intra muscular hemangioma. Even by MRI, this case was query regarding the benign nature of the lesion, the core needle biopsy was not conclusive as well. Histopathological examination confirmed the diagnosis of ESC.

As the current case revealed, the histopathological findings show cartilaginous cells with centralized zones of cellular polymorphism and proliferation of giant cells on the tumor margin. Occasionally, this tumor may present atypical morphologic characteristics, which makes the differential diagnosis with malignant lesions difficult [8].

Complete excision is a preferred mode of therapy [12]. However local recurrence rate has been reported to occur in 15–18% of the cases, therefor frequent follow up is recommended [4]. In fair of being malignant, this case underwent total excision of the mass with the surrounding normal muscles to have adequate free margins.

## 3 Conclusion

ESC is a rare benign lesion, although mostly affects the upper extremities, it can be found anywhere in the body, histopathological examination of the specimen is the diagnostic method of choice.

## Declaration of Competing Interest

There is no conflict to be declared.

## Sources of funding

No source to be stated.

## Ethical approval

Approval is not necessary for case report in our locality.

## Consent

Consent has been taken from the patient and the family of the patient.

## Author contribution

Fahmi Hussein Kakamd: Surgeon performing the operation, writing and final approval of the manuscript and follow up.

Marwan N.hassan, Karukh K.Mohammed, Dlshad R.Ahmmad, Dahat A.Hussen: Writing the manuscript, final approval of the manuscript.

Imad J Habibullah, Shvan H.Mohammed, Hiwa O. Abdulla, Diyar A.Mohammed, Abdulwahid M.Salih: literature review, final approval of the manuscript.

## Registration of research studies

Not applicable.

## Guarantor

Fahmi Hussein kakamad.

## Provenance and peer review

Not commissioned, externally peer-reviewed.
